# Comparison of BCG, MPL and cationic liposome adjuvant systems in leishmanial antigen vaccine formulations against murine visceral leishmaniasis

**DOI:** 10.1186/1471-2180-10-181

**Published:** 2010-06-24

**Authors:** Rajesh Ravindran, Sudipta Bhowmick, Amrita Das, Nahid Ali

**Affiliations:** 1 Infectious Diseases and Immunology Division, Indian Institute of Chemical Biology, 4 Raja S. C. Mullick Road, Jadavpur, Kolkata-700032, India; 2 Current Address: Department of Pathology, Emory Vaccine Center, 954 Gatewood Road, Atlanta, GA 30329, USA; 3 Current Address: Department of Zoology, Dr. Kanailal Bhattacharyya College, Dharmatala, Ramrajatala, Santragachi, Howrah-711104, India

## Abstract

**Background:**

The development of an effective vaccine against visceral leishmaniasis (VL) caused by *Leishmania donovani *is an essential aim for controlling the disease. Use of the right adjuvant is of fundamental importance in vaccine formulations for generation of effective cell-mediated immune response. Earlier we reported the protective efficacy of cationic liposome-associated *L. donovani *promastigote antigens (LAg) against experimental VL. The aim of the present study was to compare the effectiveness of two very promising adjuvants, Bacille Calmette-Guerin (BCG) and Monophosphoryl lipid A (MPL) plus trehalose dicorynomycolate (TDM) with cationic liposomes, in combination with LAg, to confer protection against murine VL.

**Results:**

All the three formulations afforded significant protection against *L. donovani *in both the visceral organs, liver and spleen. Although comparable level of protection was observed in BCG+LAg and MPL-TDM+LAg immunized mice, highest level of protection was exhibited by the liposomal LAg immunized group. Significant increase in anti-LAg IgG levels were detected in both MPL-TDM+LAg and liposomal LAg immunized animals with higher levels of IgG2a than IgG1. But BCG+LAg failed to induce any antibody response. As an index of cell-mediated immunity DTH responses were measured and significant response was observed in mice vaccinated with all the three different formulations. However, highest responses were observed with liposomal vaccine immunization. Comparative evaluation of IFN-γ and IL-4 responses in immunized mice revealed that MPL-TDM+LAg group produced the highest level of IFN-γ but lowest IL-4 level, while BCG+LAg demonstrated generation of suboptimum levels of both IFN-γ and IL-4 response. Elicitation of moderate levels of prechallenge IFN-γ along with optimum IL-4 corresponds with successful vaccination with liposomal LAg.

**Conclusion:**

This comparative study reveals greater effectiveness of the liposomal vaccine for protection against progressive VL in BALB/c. Again, evaluation of the immune responses by vaccination emphasizes the need of stimulation of potent cellular immunity based on both Th1 and Th2 cell responses to confer protection against VL.

## Background

Leishmaniases are a wide spectrum of diseases caused by trypanosomatid parasites of the genus *Leishmania *with two million new cases of human infection worldwide each year [1]. The clinico-pathological categories range from self-healing cutaneous lesions to visceral leishmaniasis (VL), the latter being an invariably fatal disease in the absence of drug treatment. Currently available chemotherapeutic agents are usually associated with high cost and toxicity [2]. Moreover, the emergence of drug resistance has raised an urgent demand for development of a safe and effective vaccine to combat the disease.

Recently, a great deal of effort has been directed towards generation of subunit vaccines that may be safer than whole cell vaccines [3]. A major limiting factor for the development of subunit vaccines is the appropriate adjuvant to enhance and tailor the effective and long lasting immune response. Bacille Calmette-Guerin (BCG) and Monophosphoryl lipid A (MPL) are two immunostimulatory adjuvants that act directly on the immune system to augment cell-mediated response to the associated antigens. BCG, in addition to being the most widely used vaccine in the world since 1921, is an immune-modulator stimulating several Toll-like receptors (TLRs) that can potentiate Th1 biased immune response [4-6]. BCG alone can protect mice against leishmaniasis [7,8], and it has also long been used as an adjuvant in field efficacy trials of candidate vaccines against leishmaniasis [9]. MPL, the non-toxic derivative of the lipopolysaccharide (LPS) of *Salmonella minnesota *is a safe and well-tolerated adjuvant approved for human use. It signals via TLR4 for the activation of T-cell effector response. Several immunization trials including *Leishmania*, malaria, human papillomavirus (HPV), Hepatitis B virus (HBV), tuberculosis and HIV with different formulations of MPL have established the safety and efficacy of this promising adjuvant [10]. Cationic liposomes are lipid-bilayer vesicles with a positive surface charge that have emerged as a promising new adjuvant technology having low toxicity and biodegradability. They are very effective antigen-deliver systems and serve to markedly enhance the uptake and presentation of antigens by antigen presenting cells. Thus, they potentiate cell-mediated and humoral immune response to poorly immunogenic protein and peptide antigens [11-14] and generate solid and durable immunity against experimental VL [15-18].

Investigations of immune protection mechanisms against leishmaniasis reveals that a shift in the balance from interleukin (IL)-4 to interferon (IFN)-γ provides the key to vaccine success in cutaneous leishmaniasis (CL) [19]. Protective immunity in VL also correlates with a Th1 and IFN- γ production [20]. But immune response to VL is a more complex reaction where an exclusive generation of a vaccine-induced Th1 is insufficient to ensure protection, and cannot predict vaccine success [21,22]. Although induction of IL-4 in infected BALB/c and noncuring models has been reported [23,24], beneficial roles of IL-4 have also been described for *L. donovani *infection [25,26].

Our earlier studies showed that leishmanial antigens (LAg) entrapped in cationic liposomes induced protection against progressive models of VL [15]. With the aim of improving vaccine formulation against this disease potential human-compatible adjuvants, BCG and MPL, were selected for combination with LAg. Thus, in the present study the protective efficacy of LAg with BCG and MPL-TDM were evaluated and compared with LAg entrapped in cationic liposomes when given by same intraperitoneal route against experimental challenge of *L. donovani *in BALB/c mice. A comparative evaluation of the immune responses elicited by the three different vaccine formulations was investigated to understand the immune mechanisms responsible for the differences in their protective abilities.

## Results

### Comparison of parasite burden in differently adjuvanted LAg vaccinated mice after *L. donovani *challenge infection

**Figure 1:**
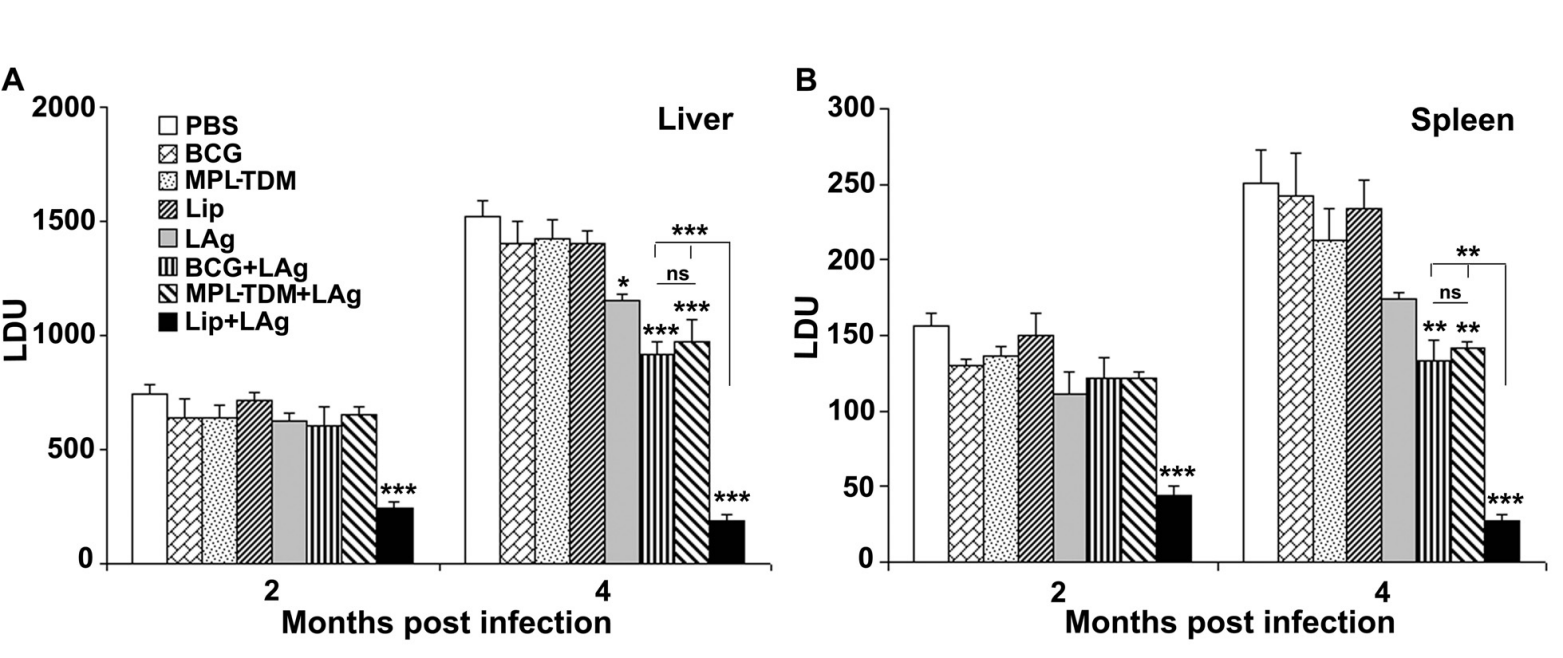
**Evaluation of protection against *L. donovani *in differently adjuvanted LAg vaccinated mice **. Kinetics of liver (A) and spleen (B) parasite burden of mice immunized intraperitoneally three times at 2-week intervals with BCG-LAg, MPL-TDM+LAg and LAg entrapped in cationic liposomes. Control animals received PBS or adjuvant only. At 10 days after the last immunization, mice were challenged intravenously with 2 × 10^7 ^promastigotes of *L. donovani*. At the designated times mice were sacrificed and LDU were calculated from the weight and microscopic examination of impression smears of liver and spleen tissues. Each bar represents the mean ± SE for five individual mice per group. The results are those from one experiment representative of two performed. Asterisks over each bar indicate significant differences in comparison to control groups. Asterisks over line indicate significant differences between groups. *, *P *< 0.05; **, *P *< 0.01; ***, *P *< 0.001; ns, not significant.

To compare the efficacy of vaccination against VL with LAg in three different adjuvants, BALB/c mice were immunized intraperitoneally with BCG + LAg, MPL- TDM+LAg and LAg entrapped in cationic liposomes. The vaccination was repeated twice at 2-week intervals and the mice were challenged intravenously with *L. donovani *promastigotes 10 days after the last immunization. Control mice received PBS or adjuvants alone. After 2 and 4 months of challenge infection clearance of hepatic and splenic parasite burden was monitored. The parasite loads were quantitated as LDU in liver and spleen biopsies. As shown in Figure 1 control mice receiving PBS or adjuvants alone developed highest parasite load in the liver and spleen as an outcome of progressive disease [15,16,27,28]. In liver, immunization with BCG + LAg and MPL-TDM + LAg did not result in any protection at 2 months post-infection (Figure 1A). However, there was significant and comparable level of decrease in parasite load in both the groups, suggesting a specific partial protection after 4 months of challenge infection as compared with PBS and corresponding free adjuvant immunized groups (*P *< 0.001). Interestingly, mice immunized with liposomal LAg showed highest reduction in parasite load in liver after 2 as well as 4 months of challenge which is significantly lower than BCG+LAg and MPL-TDM+LAg vaccinated groups (*P *< 0.001).

In BALB/c mice persistence of *L. donovani *in the spleen causes concomitant development of considerable organ-specific pathology similar to that seen in the human kala-azar. It was, therefore, important to evaluate the effect of vaccination in this organ. Similar to liver, mice immunized with BCG+LAg and MPL-TDM+LAg demonstrated partial and comparable level of protection in spleen after 4 months challenge (Figure 1B; *P *< 0.01, compared to controls). However, liposomal LAg immunization exhibited the maximum level of reduction in splenic parasite load at both 2 and 4 months after challenge (*P *< 0.001, compared to controls).

### Antigen-specific humoral responses in differently adjuvanted LAg vaccinated mice

**Figure 2:**
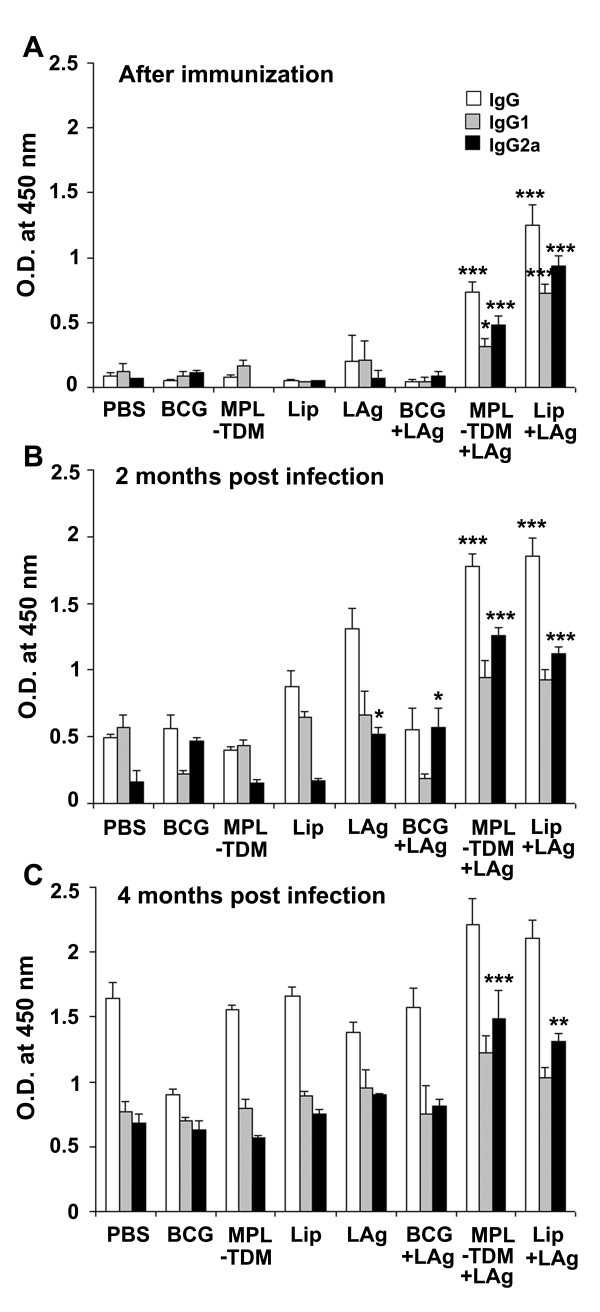
**Specific antibody responses in differently adjuvanted LAg vaccinated mice **. Mice were immunized three times at 2-week intervals. Ten days after immunization mice were challenged with *L. donovani*. Serum samples were collected after the last booster (A) and 2 (B) and 4 months (C) after infection and assayed for LAg specific IgG and its isotypes IgG1 and IgG2a antibodies by ELISA. Each sample was examined in duplicate. Each bar represents the mean absorbance values at 450 nm ± SE of five individual mice per group at designated time points. The results are those from one experiment representative of two performed. Asterisks over each bar indicate significant differences in comparison to control groups. *, *P *< 0.05; **, *P *< 0.01; ***, *P *< 0.001.

To evaluate the humoral immune responses induced by three differently adjuvanted vaccine formulations, the serum levels of leishmanial antigen-specific IgG and its isotypes, IgG1 and IgG2a, from all the vaccinated groups were assessed by ELISA. Following immunization, IgG as well as IgG1 and IgG2a were elevated in all LAg adjuvanted immunized groups, except BCG+LAg, in which they remained at background levels of control groups (Figure 2A). Higher levels of IgG, IgG1 and IgG2a were found in MPL-TDM+LAg immunized mice over the control groups (*P *< 0.05); however, the levels were low compared with liposomal LAg immunized group (*P *< 0.05). Importantly, the level of IgG2a was higher than that of IgG1 in both MPL-TDM+LAg and liposomal LAg immunized mice. With progressive infection, significant increase in total IgG was detected in all the immunized groups that became comparable to controls after 4 months of challenge infection (Figure 2B and 2C). Increased levels of IgG2a were still maintained in MPL-TDM+LAg and liposomal LAg immunized groups compared to control groups (*P *< 0.01).

### Stimulation of DTH response in differently adjuvanted LAg vaccinated mice

**Figure 3:**
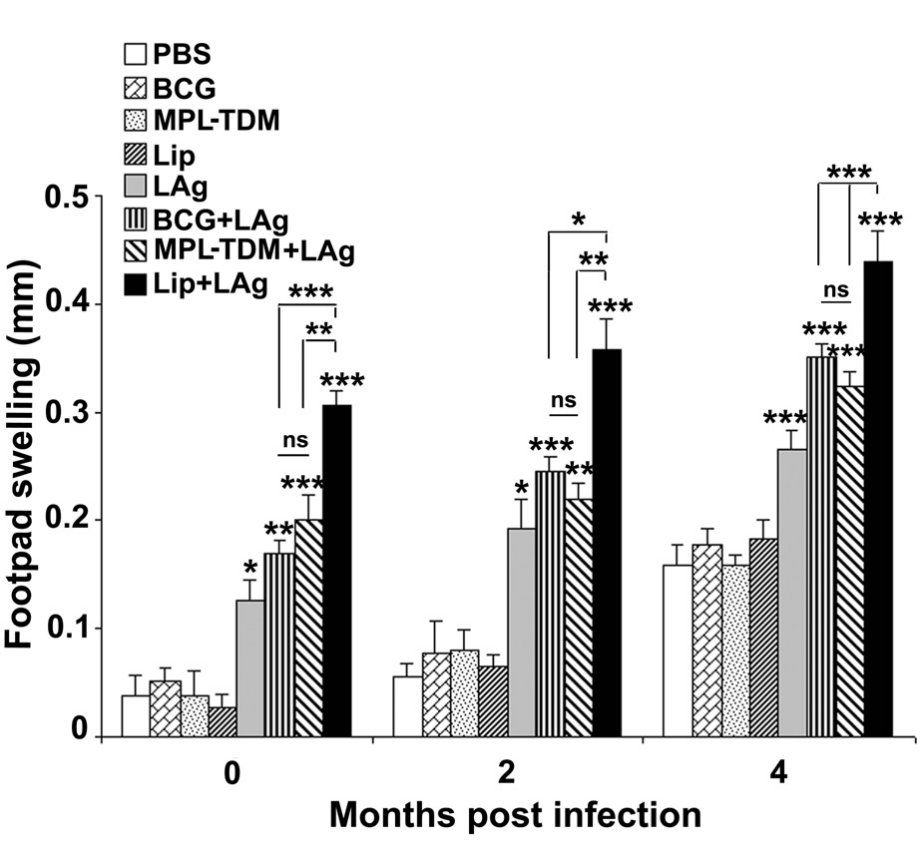
**DTH responses in differently adjuvanted LAg vaccinated mice **. Mice were immunized three times at 2-week intervals. Ten days after immunization mice were challenged with *L. donovani*. After the last immunization and 2 and 4 months after infection LAg-specific DTH responses were measured. The response is expressed as the difference (in mm) between the thickness of the test (LAg-injected) and control (PBS-injected) footpads at 24 h. Each bar represents the mean ± SE for five individual mice per group at designated time points. The results are those from one experiment representative of two performed. Asterisks over each bar indicate significant differences in comparison to control groups. Asterisks over line indicate significant differences between groups. *, *P *< 0.05; **, *P *< 0.01; ***, *P *< 0.001; ns, not significant.

As an index of parasite antigen specific cell mediated response in vivo, DTH response was measured in vaccinated mice 10 days after last immunization and recalled at 2 and 4 months after challenge infection. Vaccinated mice with free LAg and its combination with different adjuvants displayed significant DTH response in comparison to control groups (Figure 3; *P *< 0.05). However, the response by both BCG and MPL-TDM adjuvanted LAg was comparable but lower than the response induced by liposomal LAg immunization (*P *< 0.01). With challenge infection the response was increased progressively in LAg and its adjuvanted immunized groups and showed that the levels were significantly higher compared to the control groups at 2 and 4 months post-infection (*P *< 0.05). Among the differently adjuvanted groups, BCG+LAg and MPL-TDM+LAg immunized mice exhibited comparable levels of response whereas higher response was induced by the liposomal LAg immunized group (*P *< 0.05) at all time points after challenge infection.

### Generation of IFN-γ and IL-4 response in differently adjuvanted LAg vaccinated mice

**Figure 4:**
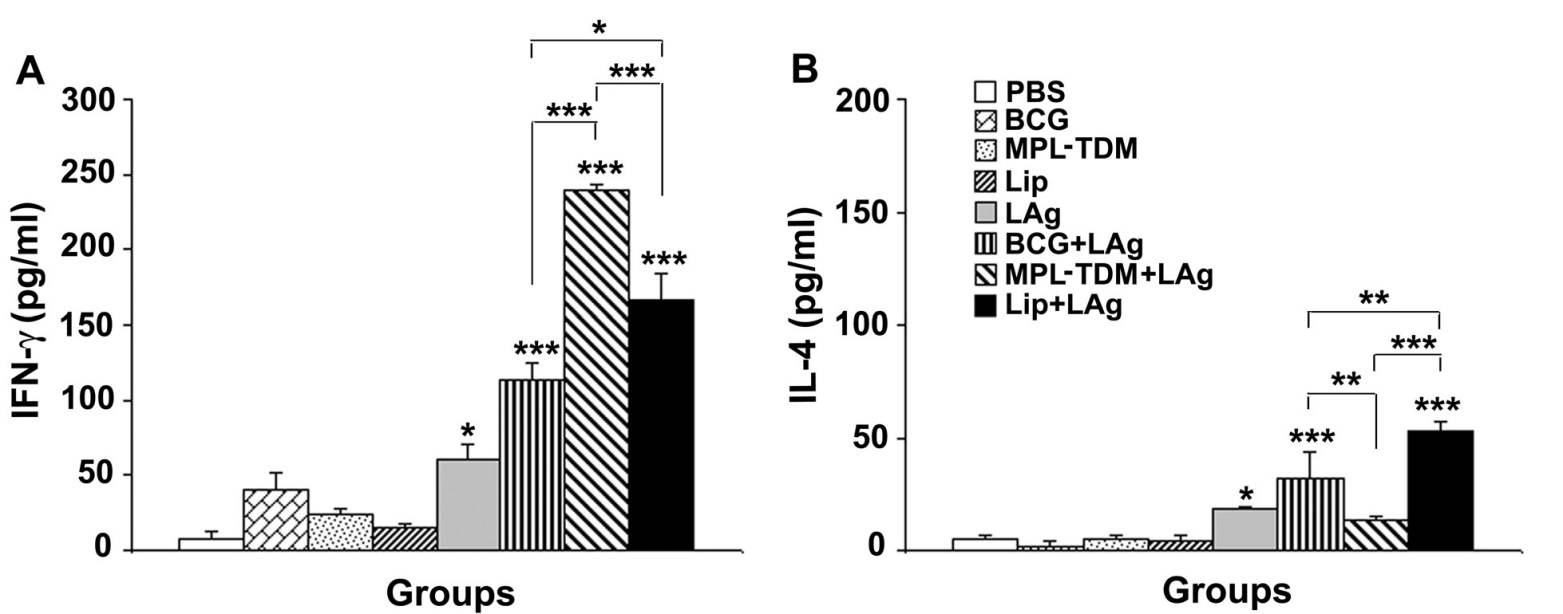
**IFN-γ and IL-4 responses in differently adjuvanted LAg vaccinated mice **. Mice were immunized three times at 2-week intervals. Ten days after last immunization spleens were collected from mice and restimulated in vitro with LAg (10 μg/ml). After 72 h supernatants were collected and concentrations of released IFN-γ (A) and IL-4 (B) levels were determined by ELISA. Each sample was examined in duplicate. Each bar represents the mean ± SE for five individual mice per group. The results are those from one experiment representative of two performed. Asterisks over each bar indicate significant differences in comparison to control groups. Asterisks over line indicate significant differences between groups. *, *P *< 0.05; **, *P *< 0.01; ***, *P *< 0.001.

Although BCG+LAg failed to induce serological response after immunization, the response was enhanced with infection and become comparable with other groups. Conversely, BCG+LAg and MPL-TDM+LAg immunization induced and maintained comparable level of cell-mediated immune response with challenge infection which led to protection in both the groups. Thus investigation of detailed vaccine induced cell-mediated response after immunization may help to understand the underlying mechanism of different formulations that can correlate with the observed protection. Next, we evaluated the Th1 and Th2 cytokine responses in differently adjuvanted mice. Splenocytes from immunized mice were isolated 10 days after immunization and, IFN-γ and IL-4 levels were measured in vitro following restimulation with LAg. LAg in different adjuvant vaccinated groups produced substantial amounts of IFN-γ compared to controls (Figure 4A; *P *< 0.001). Interestingly, the most pronounced increase in IFN-γ level was observed in MPL-TDM+ LAg vaccinated groups in comparison to other groups (*P *< 0.001). Mice immunized with BCG+LAg secreted lower amount of IFN-γ compared with the liposomal LAg immunized group (*P *< 0.05). Mice receiving BCG+LAg and liposomal LAg immunization showed significant increase in IL-4 production compared to controls (Figure 4B, *P *< 0.001). However, elicitation of significantly higher IL-4 response was observed in liposomal LAg vaccinated mice compared to BCG+LAg immunized groups (*P *< 0.01). In contrast to the robust IFN-γ responses observed with MPL-TDM+LAg vaccine, IL-4 level was significantly lower from other vaccinated groups (*P *< 0.01). Thus, MPL-TDM+LAg triggered highest IFN-γ but lowest IL-4 indicating an exclusive Th1 cell-mediated immune response. BCG+LAg and liposomal LAg generated a mixed Th1/Th2 response as evident from significant production of both IFN-γ and IL-4 post-immunization groups. But compared to the Th1/Th2 response generated by liposomal LAg, the cytokine levels were lower for BCG+LAg immunized groups.

## Discussion

Despite the current knowledge of immunology and pathology related to the parasite *Leishmania*, till now, a desirable vaccine for humans has not been successfully developed. The main goal of vaccination is the induction of a protective immune response against the pathogen. Successful vaccination strategies for *Leishmania *have relied on presentation of antigen with appropriate adjuvants to the host immune system to stimulate effective cell-mediated immune responses. The present study is the first direct, head-to-head comparison of vaccine formulations using three different adjuvants, BCG, MPL-TDM and cationic liposomes, with the same leishmanial antigen for their efficacy against *L. donovani *challenge in BALB/c model.

BCG and MPL were chosen as adjuvants in this study as they are human-compatible potent inducer of cell-mediated immunity. BCG, being almost the only adjuvant licensed for human use and effective against intracellular pathogen infections, was extensively used in clinical trials of vaccination against CL and VL [9]. Amongst the adjuvants recently approved for human vaccines is MPL, a potent stimulator of Th1 response, being evaluated in clinical trials against various diseases including malaria, tuberculosis and leishmaniasis [10]. Previous studies from our laboratory established that cationic liposomes is a potent adjuvant as they have the ability to enhance protective cell-mediated immune response against experimental VL [15-18]. Thus, cationic liposomes was selected to compare its efficacy with two other human-compatible adjuvants BCG and MPL to confer protection against *L. donovani *infection.

Comparison of the vaccine potentiality of cationic liposomal formulation of LAg with BCG+LAg and MPL-TDM+LAg revealed that all the three vaccines afforded significant protection against challenge with *L. donovani*. However, cationic liposome was the most potent of the three adjuvants and conferred protection superior to other two adjuvants. The ability of cationic liposomes to induce significant protection with LAg is entirely consistent with results of our previous studies in mice as well as hamster models of VL [15]. However, the level of protection afforded by this formulation was lower than mice immunized with SLA (soluble leishmanial antigens) entrapped in these vesicles or LAg entrapped in neutral and cationic DSPC liposomes [16,27,29], suggesting entrapment of more immunogenic antigens or optimization of liposomal formulation could influence the efficacy of cationic liposomes. Cationic liposomes was also shown to be a potent adjuvant to enhance immune response against CL [30]. BCG is the most widely used adjuvant in clinical vaccine trials against leishmaniasis including VL. Although the vaccines were found to be safe and immunogenic, the efficacy was not carried over to a protective effect [31,32]. Reports on the ability of BCG-vaccine to protect against leishmaniasis even in experimental models vary from effective [33,34] to partial protection [35,36]. MPL-SE (stable emulsion) has been found to be safe and efficacious against cutaneous and mucosal leishmaniasis in mice, non-human primates and humans when vaccinated with *Leishmania*-derived recombinant polyprotein Leish-111f or its component proteins [37-39]. In experimental model of VL, MPL-SE formulated Leish-111f was effective in reducing splenic parasite burden [37] whereas recombinant sterol 24-c-methyltransferase (rSMT) plus MPL-SE afforded significant protection in both liver and spleen [40]. Furthermore, although MPL formulated 78 kDa antigen of *L. donovani *was efficacious in liver against challenged with *L. donovani *infection [41], partial protection was observed with *Leishmania *antigen in association with MPL-Dimethyl dioctadecylammonium bromide (DDA) in spleen [42], an organ where parasites persist and are more resistant to various immunological interventions and even T cell-dependent chemotherapy.

Serological data show that mice vaccinated with MPL-TDM+LAg and liposomal LAg induced strong humoral responses after immunization that persisted after challenge infection. Conversely and in accordance to previous reports [33,34], mice vaccinated with BCG-LAg failed to respond with the production of antibodies prior to infection. BCG is known to stimulate APCs through several TLRs as well as to activate and recruit NK cells and neutrophil granulocytes. However, it could not act as a depot for coadministered antigens for generation of antibody response [43].

Successful vaccination for the control of parasite multiplication is often related to antigen induced DTH response as an indication of activation of cell-mediated response. In the present study, results obtained upon vaccination with LAg in association with BCG, MPL-TDM and liposomes demonstrated induction of an appreciable DTH response suggesting the activation of cell-mediated immunity. The induction of DTH was, however, highest in mice immunized with liposomal LAg with lower and comparable levels induced by BCG+LAg and MPL-TDM + LAg. In clinical trials injection of BCG mixed with killed parasites significantly increased cell-mediated immune responses to the vaccine was measured by leishmanin skin test (LST). The LST conversion due to vaccination corresponded with reduced incidence of infection at least in the subpopulation of "responders" to vaccination [32]. Animals successfully vaccinated with BCG and leishmanial antigens similarly elicited DTH reactions [33,34]. Significant elevation of DTH response in mice immunized with protein antigens and MPL-DDA that provided resistance against VL has also been reported [42]. The significantly higher DTH response induced by liposomal LAg over BCG+LAg and MPL-TDM+LAg before and after challenge infection demonstrates elicitation of strong and persistent cell-mediated immunity by this vaccine, which resulted in greater resistance against disease.

An important leishmanicidal effector mechanism is the production of IFN-γ by *Leishmania*-specific cells, which in turn activates macrophages to kill intracellular parasites. Immunization of BALB/c mice with BCG, MPL-TDM and liposomal LAg resulted in high IFN-γ production following *in vitro *restimulation. The levels of IFN-γ, however, varied in the three vaccination groups. Moderate levels of IFN-γ were produced by liposomal vaccine followed by BCG+LAg vaccine. In contrast, robust levels of IFN-γ were observed with MPL-TDM+LAg vaccine. Interestingly, whereas immunization with liposomal as well as BCG+LAg also led to very significant, though variable, levels of IL-4 production, the level of IL-4 by MPL-TDM+LAg vaccine was low. A Th1 phenotypic response was thus elicited by MPL-TDM+LAg whereas liposomal and BCG+LAg elicited a mixed Th1/Th2 response. IFN-γ, a signature cytokine of Th1 response is associated with resistance against *L. major*. But high IFN-γ production cannot be the sole criterion that might confer protection against *L. donovani *[19]. Moreover, in contrast to CL, early IL-4 production is not detrimental and may have a protective role in VL [16-18,25,27]. The role of IL-4 in conferring protection against *L. donovani *is also supported from a finding where chemotherapy against VL in IL-4 ^-/- ^mice is not effective [26]. Thus, the optimum levels of both the cytokines IFN-γ and IL-4 induced by the liposomal LAg vaccination substantiate earlier observations that a mixed Th1/Th2 response is essential for protection against VL [16-18,27,44]. Hence, we believe that the inability of MPL-TDM to stimulate optimal IL-4, as observed with the liposomal vaccine formulation, is probably the major factor for its partial success in protection. The low immunogenecity of BCG+LAg characterized by sub-optimal antigen-specific IFN-γ and IL-4 responses may be responsible for the low level of protection induced by this vaccine.

In order to compare the protective efficacy of BCG and MPL-TDM with liposome, all the three vaccine formulations were administered through the intraperitoneal route. In contrast to liposomes, the success or failure of protection with BCG+LAg and MPL-TDM+LAg was probably not dependent on the route of immunization. Although, intradermal route of immunization is favoured for BCG formulations, intraperitoneal vaccination of BCG with a combination of dehydroepiandrosterone peptide has been reported for the successful prevention of asthma development [45]. Again, subcutaneous administration of MPL vaccine has been found to be successful for vaccinination against leishmaniasis [37]. Further, immunization of MPL-TDM in association with an immunogenic peptide administered either through subcutaneous or intraperitoneal routes was found to induce the same Th1-biased response [46]. Conversely, administration of liposomal LAg through subcutaneous route failed to induce protection in experimental mice model of VL [47]. When the intraperitoneal route is used, peritoneal macrophages are the major population of APCs available. It has been found that induction of the immune response by liposomal delivery of antigen is mainly macrophage dependent and DCs are considered to be less efficient in phagocytosis than cells of the macrophage lineage [48]. Thus intraperitoneal immunization of liposomal antigen could effectively generate a protective immune response. Since BCG and MPL-SE have been used for intradermal, subcutaneous or intramuscular injection and may not be optimal for intraperitoneal injection, their responses with LAg through one of these routes could help in conclusive comparison of liposomes. Further, since MPL is a potent inducer of Th1 response and can function through subcutaneous route also, we speculate that MPL can be combined with liposomes and can be administered through subcutaneous route to overcome the failure of liposomal vaccine through this route. Indeed we have preliminary evidence showing that immunization with liposomal antigens in association with MPL-TDM can induce protection against *L. donovani *infection in BALB/c mice through subcutaneous route (unpublished observation). AS01, a liposomal formulation containing MPL as a potent inducer of humoral and cell-mediated response is already in clinical trials for malaria [10]. Thus liposomal formulated MPL-TDM+LAg may be the choice of adjuvant for vaccine development against *Leishmania *and other intracellular pathogens.

## Conclusions

This comparative study of BCG+LAg and MPL-TDM + LAg vaccines with cationic liposomal formulation of LAg interestingly reveals a significantly greater effectiveness of the liposomal vaccine for protection against progressive VL in BALB/c. Evaluation of the immune responses emphasize the need for an immunogenic vaccine for elicitation of potent vaccine-induced cellular immunity based on both Th1 and Th2 cell responses to confer protection against the visceral disease. Thus, the cationic liposomes offer a rational choice of adjuvant for the development of vaccines against a range of infectious diseases such as leishmaniasis, malaria and tuberculosis.

## Methods

### Animals

Female BALB/c mice (4-6 weeks old), bred in the animal facility of Indian Institute of Chemical Biology (Kolkata), were used for experimental purposes with approval of the IICB Animal Ethical Committee and mice were handled according to their guidelines.

### Parasites and culture condition

*L. donovani*, strain AG83 (MHOM/IN/1983/AG83) was originally isolated from an Indian kala-azar patient and maintained in Syrian golden hamsters by serial passage as described elsewhere [15]. Briefly, promastigotes were grown at 22°C in Medium 199 (pH 7.4) supplemented with 20% heat inactivated fetal bovine serum (FBS), 2 mM L-glutamine, 100 U/ml penicillin, 25 mM HEPES, 100 μg/ml streptomycin sulphate (all from Sigma-Aldrich, St. Louis, USA), and the parasites were subcultured in the same medium at an average density of 2 × 10^6 ^cells/ml at 22°C [15].

### Preparation of leishmanial antigens

LAg was prepared from *L. donovani *promastigotes as described earlier [15]. Briefly, stationary phase promastigotes, harvested after the third or fourth passage in liquid culture, were washed four times in cold 20 mM phosphate-buffered saline (PBS), pH 7.2, and resuspended at a concentration of 1.0 g cell pellet in 50 ml of cold 5 mM Tris-HCL buffer (pH 7.6). The suspension was vortexed six times at 2 min each with a 10-min interval on ice and centrifuged at 2,310 × *g *for 10 min. The crude ghost membrane pellet thus obtained was resuspended in the same Tris buffer and sonicated three times for 1 min each at 4°C in an ultrasonicator (Misonix, New York, USA). The suspension was finally centrifuged for 30 min at 5,190 × *g*, and the supernatant containing leishmanial antigens (LAg) was harvested and stored at -70°C until used. The amount of protein obtained from a 1.0 g cell pellet was approximately 14 mg, as assayed by the method of Lowry et al. [49] with bovine serum albumin as the standard, in the presence of 1% sodium dodecyl sulphate and appropriate blanks.

### Adjuvants

Positively charged liposomes were prepared with egg lecithin, cholesterol, and stearylamine (7:2:2 molar ratio), respectively as reported earlier [15]. MPL (0.5 mg) plus trehalose dicorynomycolate (TDM) (0.5 mg) in 2% oil (squalene)-Tween 80-water was purchased from Sigma-Aldrich Corp., St. Louis, USA. Briefly, each vial was reconstituted with 1 ml saline and mixed at 1:1 ratio with LAg in PBS and administered in mice as 50 μg/dose. The mean particle size of the emulsion droplets was 128 ± 6.65 as determined by Zetasizer Nano-ZS (Malvern Instruments, Worcestershire, UK). Bacillus Calmette Guerin (BCG) (Pasteur Institute, Paris, France) was diluted in PBS mixed at 1:1 ratio with LAg in PBS prior to injection to an administrable dose of 5 × 10^4 ^cells/mice.

### Entrapment of leishmanial antigens into cationic liposomes

For encapsulation of the LAg in the liposomal vesicles the lipid film was dispersed in PBS containing 1 mg/ml LAg and sonicated for 30 s in an ultrasonicator (Misonix) [15]. Liposomes with entrapped LAg were separated from excess free materials by three successive washing in PBS with ultracentrifugation (105,000 × g, 60 min, 4°C). The mean size of the LAg entrapped liposomes was 337.3 ± 10.2 as determined by Zetasizer Nano-ZS (Malvern Instruments). The presence of antigen could not influenced the size of the vesicles (empty vesicles mean size 306.8 ± 2.6). The protein content entrapped into liposomes was estimated by the method described by Lowry et al. [49]. The phospholipid content of liposomes was 15.5 mg/ml as determined using the Stewart assay [50]. The average amount of LAg associated per mg of egg lecithin was 33 μg.

### Vaccination and challenge infection

BALB/c mice were vaccinated by three intraperitoneal injections of 20 μg of free LAg, incorporated in liposomes, or associated with other adjuvants at 2-week intervals in a total volume of 200 μl. PBS and only adjuvant treated animals were included as controls. Ten days after last immunization the animals were challenged intravenously with 2 × 10^7 ^freshly transformed promastigotes [15].

### Evaluation of infection

At the times designated in Results, the course of infection was evaluated by microscopic examination of Giemsa-stainted impression smears of liver and spleen samples. The organ parasite burden was expressed as Leishman-Donovan units (LDU) calculated as follows: number of amastigotes per 1,000-host cell nuclei × organ weight (in mg) [51].

### Antigen-specific antibody responses by ELISA

For determination of antibody responses, serum samples collected from experimental groups of mice before and after infection were analyzed for the presence of LAg-specific immunoglobulin by ELISA. 96 well microtitration plates (maxisorp plates; Nunc, Roskilde, Denmark) were coated with 100 μl of LAg (25 μg/ml) diluted in 20 mM phosphate buffer (pH 7.5) overnight at 4°C. Non-specific binding sites were blocked with 1% bovine serum albumin (BSA) in PBS at room temperature for 3 h. After washing with PBS containing 0.05% Tween-20 (Sigma-Aldrich), the plates were incubated overnight at 4°C with 1:1000 dilutions of mice sera. The plates were then washed and incubated with horseradish peroxidase-conjugated goat anti-mouse IgG (Sigma-Aldrich) diluted 1:5000 and antimouse IgG1 or IgG2a (BD Pharmingen, San Diego, USA) diluted 1:1000 in blocking buffer. Finally, colour reaction was developed by the addition of 100 μl/well of substrate solution (*o*-phenylene diamine dihydrochloride, 0.8 mg/ml in 0.05 M phosphate-citrate buffer, pH 5.0, containing 0.04% H_2_O_2_) for 30 min. Absorbance was determined at 450 nm using ELISA plate reader (Thermo, Waltham, USA) [15].

### Delayed type hypersensitivity (DTH)

After the last vaccination, 2 and 4 months after challenge infection, delayed-type hypersensitivity (DTH) was determined as an index of cell-mediated immunity. The response was evaluated by measuring the difference in the footpad swelling at 24 h following intradermal inoculation of the test footpad with 50 μl of LAg (800 μg/ml) from that of control (PBS- injected) footpad with a constant pressure caliper (Starret, Anthol, USA) [15].

### Cytokine Assay

Spleens were removed aseptically from experimental mice of each group at 10 days after last immunization and teased between 20 μm pore size sieve into single cell suspension in complete medium prepared with RPMI 1640 supplemented with 10% FBS, 10 mM NaHCO_3, _10 mM HEPES, 100 U/ml penicillin, 100 μg/ml streptomycin sulphate, and 50 μM β-mercaptoethanol (Sigma-Aldrich). Erythrocytes were removed by lysis with 0.14 M Tris buffered NH_4_Cl. The splenocytes were washed twice, resuspended in culture medium and viable mononuclear cell number was determined by Trypan blue exclusion. Splenocytes were then cultured in a 96-well flat-bottomed ELISA plate (Nunc) at a density of 2 × 10^5 ^cells/well in a final volume of 200 μl. The cells were restimulated *in vitro *with medium alone or with LAg (10 μg/ml) and supernatants were collected after 72 h incubation at 37°C in a humified chamber containing 5% CO_2 _and stored at -70°C until use. Measurements of IFN-γ and IL-4 concentrations were carried out using Opt EIA Kits (BD Pharmingen) as detailed in manufacturers' instructions [27].

### Statistical analysis

One-way ANOVA statistical test was performed to assess the differences among various groups. Multiple comparisons Tukey-Kramer test was used to compare the means of different experimental groups. A value of *P *< 0.05 was considered to be significant.

## Abbreviations

VL: Visceral leishmaniasis; LAg: *L. donovani *promastigote antigens; BCG: Bacille Calmette-Guerin; MPL: Monophosphoryl lipid A; TDM: Trehalose dicorynomycolate

## Authors' contributions

RR performed all the experiments of this study. SB and NA have contributed in designing of the paper. SB and AD wrote the draft of the manuscript. NA conceived the study, coordinated it and revised the manuscript. All authors read and approved the final manuscript.
